# Chemotherapeutic potential of Cayratia trifolia L nhexane extract on A2780 cells

**DOI:** 10.6026/97320630017710

**Published:** 2021-08-31

**Authors:** Chella Perumal Palanisamy, Sivakumar Pethanan, Gopinath Gurulingam Vincent, Karthigeyan Murugesan, Ananthavalli Ramachandran, Ramu Sivanandam, Mani Panagal

**Affiliations:** 1 State Key Laboratory of Biobased Material and Green Papermaking, School of Food Science and Engineering, Qilu University of Technology, Shandong Academy of Science, Jinan 250353, China; 2 Department of zoology, M R Government arts college, Mannargudi, Tamil Nadu - 614001, India; 3 Department of zoology, Arumugam Pillai Seethai Ammal College, Tiruppathur, Tamil Nadu, India; 4 Department of Biotechnology, Annai College of Arts and Science, Kovilacheri, Kumbakonam, Tamil Nadu - 612503, India

**Keywords:** *Cayratia trifolia*, CXCR4 and HER2 protein expression, Metastatic signalling, Anti-ovarian cancer activity

## Abstract

It is of interest to report the chemotherapeutic (drug target based) potential of n-hexane Cayratia trifolia L. (C.trifolia) extract on A2780 cell lines. mRNA and protein expression analysis of the human chemokine receptor (CXCR4) and human epidermal growth factor receptors-2 (HER2) were studied using RT-PCR analysis and western blot analysis. The results show significant cell growth inhibition with minimal IC50 values of 46.25 ± 0.42 micro g/mL against A2780 cell lines. mRNA and protein expression were considerably reduced in *C. trifolia*treated A2780 cell lines for further consideration as a chemotherapeutic agents.

## Background:

The frequent use of synthetic drugs cause numerous side effects with drug resistance [1]. Natural products are effective in reducing the toxicity of allopathic drugs and therapy [2]. It is known that secondary metabolites possess strong antioxidant, cytotoxicity, antimicrobial, antidiuretic, antidiabetic, anti-inflammatory activities and also used to treat other disease and disorders, hence, play a major role in the management of human disease [3]. Cayratia trifolia L. (C. trifolia, Family: Vitaceae) commonly referred to as fox grape in English, is native to India, Australia and few Asian countries [4]. The presence of yellow waxy oil, steroids, terpenoids, alkaloids, flavonoids such as kaempferol, myricetin, quercetin, triterpenes, epifriedelanol and tannins in the whole plant of C. trifolia is known [5-7]. The paste of the tuber from C. trifolia is used in the treatment of snake bite [9].

We have shown the antioxidant, antimicrobial and cytotoxicity potential of n-hexane extract of Cayratia trifolia L [8]. Therefore, it is of interest to explore that chemotherapeutic (drug target based) potential of Cayratia trifolia L n-hexane extract on A2780 cells through suppression of mRNA expression.

## Materials & Methods:

### Plant collection and authentication:

The whole plant of C. trifolia was collected from in the campus of Annai College of Arts and Science, Kovilacheri, Kumbakonam, Thanjavur District, Tamil Nadu, India and the plant was authenticated by Dr. P. Sathyanarayanan, Botanical survey of India, TNAU Campus, Coimbatore (voucher number is BSI/SRC/5/23/2010-2011/Tech.1527). The plant material was shade dried, powdered and stored in air tight container at 4°C for future analysis [10].

### Extract preparation:

The dried plant material was subjected to n-hexane extraction using exhaustive extraction procedure [11]. Briefly, 200g of the plant material was soaked in a flask containing 1000 mL of n-hexane and was kept on the rotating shaker for 72 hours at 25°C (average room temperature). Finally, the collected extract was concentrated through rotary evaporator (RE-2A evaporator) set at 40°C. Further, it was stored at 4°C for future studies.

### Cytotoxicity analysis:

The cytotoxicity assay was determined by MTT assay [12]. Briefly, 5000 cells were seeded in each well on 96 well plates and cultured for 24 hours, then treated with different concentration (3.12, 6.25, 12.5, 25, 50, 100, 200 µg/mL) of plant extract while cyclophosphamide was used as positive control. The cells were then incubated at 37°C for 24 hours in 5% CO2. At the end of the incubation, the medium was removed and 10 µL of MTT was added followed by 100 µL of DMSO was added to each well to solubilize the formazan crystals. It was then left in dark at room temprature. The absorbance was measured at the wavelength of 595 nm using a mircotitre plate reader and the results were analysed in triplicate and the percentage was calculated. This related procedure is taken from our previous work [8].

### Gene expression analysis:

Total RNA isolation, cDNA conversion and real-time PCR:

mRNA expression levels of CXCR 4; HER2 were examined using real-time PCR. The total RNA was isolated by using using a TRIR kit (Total RNA Isolation Reagent Invitrogen)and estimated spectrometrically by the method of Laneve et al. (2014) [13]. The RNA concentration was expressed in microgram (µg). By using the reverse transcriptase kit from Eurogentec (Seraing, Belgium), complementary DNA (cDNA) was synthesized from 2 µg of total RNA as stated in the manufacturer's protocol. To perform real-time PCR, the reaction mixture containing 2x reaction buffer (Takara SyBr green master mix), Forward and reverse primers of CXCR4 and HER2 (the primer sequences were listed in Table 1 - see PDF) in total volume of 45 µl expect the cDNA was made, mixed intensively and spun down. In individual PCR vials, about 5 µl of control DNA for positive control, 5 µl of water for negative control and 5 µl of template cDNA for samples were taken and reaction mixture (45 µl) were added. 40 cycles (95°C for 5 min, 95°C for 5 s, 60°C for 20 s and 72°C for 40 s) was set up for the reaction and obtained results were plotted by the PCR machine (CFX96 Touch Real-Time PCR Detection System, USA) on a graph. Relative quantification was calculated from the melt and amplification curves analysis.

### Protein expression analysis by western blotting:

After the 24 h treatment period the cells were lysed in RIPA buffer containing 1X protease inhibitor cocktail, and protein concentrations were determined by Lowry's method [14]. Cell lysates (50 µg) was subjected to heat denaturation at 96°C for 5 min with Laemmli buffer. Proteins were resolved by sodium dodecyl sulfate polyacrylamide gel electrophoresis (SDS-PAGE) on 12% polyacrylamide gels and then transferred to PVDF membrane (Amersham Biosciences, UK). The membrane was blocked with 5% blocking buffer (Amersham Biosciences, UK) in TBS-T (Tris buffered saline and Tween 20), for 1 h at room temperature followed by incubation with primary antibody to CXCR4 and HER2 at a dilution of 1:1000. The membrane was subjected to repeat wash for three times with TBS- T and then incubated for 1 h in horseradish peroxidase (HRP)-conjugated mouse/rabbit secondary antibody by 1:7500 dilutions in TBS-T. The membrane was again subjected to repeated wash for three times with TBS and TBS-T. Protein bands were visualized in chemidoc using enhanced chemiluminescence reagents (ECL; Amersham Biosciences, UK). The detected bands were quantified using the Quantity Software (Bio-Rad). Later, the membranes were incubated in stripping buffer [50 ml, containing 62.5 mM of Tris-HCl (pH 6.7) and 1 g of SDS and 0.34 ml of β-mercapto ethanol] at 55°C for 40 min. Following this, the membranes was reprobed using β-actin antibody (1:5000). In this study, β-actin was used as the loading control.

### Statistical analysis:

The obtained results from the assays were showed as mean ± SD. The Statistical evaluations were measured through statistical package program (SPSS 10.0, IBM, Armonk, New York, United States).

## Results and Discussion:

**Figure 1:**
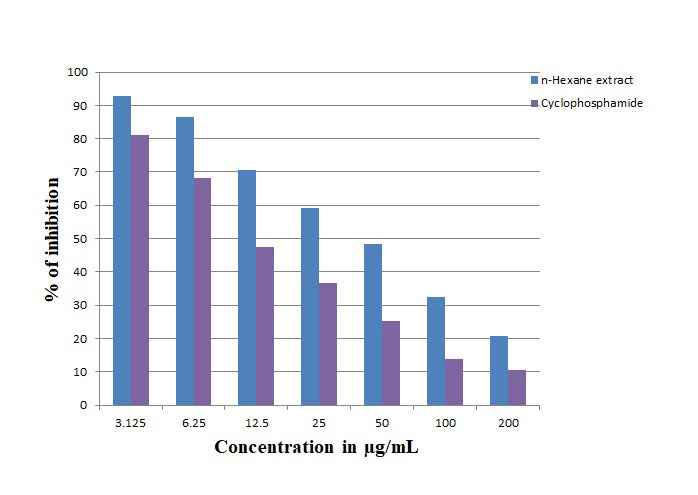
Cell growth inhibitory assay as reproduced from elsewhere [8]

The in vitro cytotoxicity activity of n-hexane extract of C.trifolia was investigated using different concentrations ranging from 3.12 to 200 µg/mL against A2780 ovarian cancer cell lines. n-hexane extract showed 86% of cell growth inhibition at the highest concentration of 200 µg/mL, where as cyclophosphamide showed 88% ie, significant cell growth inhibitory activity (IC 50 value) by n-hexane extract (Figure 1) was observed to be 46.25±0.42µg/mL, when compared to the standard, cyclophosphamide. The present study has confirmed that the induction of cell death occured at a very low concentration like any other potential cytotoxicity drug. Thus, it may be considered to be a good candidate for therapeutic agent.

A part of the chemokine superfamily, chemokine receptor 4 (CXCR4) is a particular stromal cell factor-1 (SDF-1, CXCL12) receptor, which is a strongly conserved G protein-coupled 7-transmembrane receptor. The only chemokine receptor expressed in about 80% of ovarian cancer tissues, although not in the usual ovarian epithelium, is CXCR4, rendering it a potential candidate for targeted ovarian cancer therapy [15]. HER2 was, on the other hand, a significant predictor of oncogenic genes in ovarian cancer. Breast cancer studies have shown that HER2 controls CXCR4 expression, which is essential for HER2-enhanced lung invasion, migration, adhesion and metastasis [16]. The X-ray structure of the bioactive compound epifriedelanol, isolated from the ethanol extract C.trifolia and its binding affinities to a few proteins identified to be overexpressed under ovarian cancer (HER2, EGFR and CXCR4) have been extensively investigated using molecular docking techniques. It is very impressive that the compound binds to carboplatin, an FDA-approved treatment for ovarian cancer, in order to interact with the protein targets [17]. Therefore in the present study, we attempted to investigate the underlying signalling mechanism of C.trifolia in A2780 ovarian cancer cells. Results of this study showed that CXCR4 (p<0.036) mRNA and CXCR 4 protein (p<0.015) expression were significantly reduced in C.trifolia treated A2780 cells (Figure 2 - see PDF). Similar to that of the CXCR4, HER2 mRNA (p<0.022) and protein (p<0.007) were also down regulated in extract treated cells. Our findings showed that CXCR4 and protein expression was substantially up regulated by HER2 in untreated A2780 cells (Figure 3 - see PDF). Treatment with C.trifolia n-hexane extract controlled the HER2 mRNA and protein expression which in turn down regulated the CXCR4 expression. These data shows that natural compounds selected from the GC-MS study of C.trifolia ethanolic extract, such as ethyl oleate, 4,8,12,16-tetramethylheptadecan-4-olide and heptacosanol, have strong HER2 molecule docking and have an appropriate score and complex energy compared to the FDA-approved cyclophosphamide drug [16] and this may be the reason behind the down regulation of HER2 and CXCR4 by n-hexane C.trifolia extract that regulates ovarian cancer.

## Conclusion:

The n-hexane extract of C.trifolia significantly reduced CXCR4 and HER2 mRNA and protein expression A2780 cell lines. However, role of C.trifolia n-hexane extract on further downstream signalling molecules need to be studied to validate the data.

## References

[1] Mohammed SI (2017). Indian J Clin Biochem..

[2] Priyanga S (2015). Der Pharmacia Lettre..

[3] https://innovareacademics.in/journals/index.php/ijpps/article/view/10295.

[4] Perumal PC (2012). Asian Pac J Trop Dis..

[5] Sowmya S (2015). Indo Am J Pharm Res..

[6] Perumal PC (2015). Pharmacognosy Res..

[7] Sowmya S (2015). Int J Toxicol Pharm Res..

[8] Bhuvaneswari M (2021). Bioinformation..

[9] Perumal PC (2014). J App Pharm Sci..

[10] Sowmya S (2014). World J Pharm Res..

[11] Palanisamy CP (2019). S Afr J Bot..

[12] Ngamwongsatit P (2008). J Microbiol Methods..

[13] Laneve P (2014). J Vis Exp..

[14] Lowry OH (1951). J Biol Chem..

[15] Palanisamy CP (2018). J Young Pharm..

[16] Palanisamy CP (2019). J Pharm Bioallied Sci..

[17] https://www.banglajol.info/index.php/BJP/article/view/24933/1845.

